# The impact of immunosuppression on postoperative graft function after graft-unrelated surgery: a retrospective controlled cohort study

**DOI:** 10.1186/s12882-019-1358-2

**Published:** 2019-05-16

**Authors:** Ann-Kathrin Lederer, Dominic Haffa, Philipp Felgendreff, Frank Makowiec, Stefan Fichtner-Feigl, Roman Huber, Lampros Kousoulas

**Affiliations:** 1 Center for Complementary Medicine, Institute for Infection Prevention and Hospital Epidemiology, Medical Center – University of Freiburg, Faculty of Medicine, University of Freiburg, Breisacher Straße 115b, 79106 Freiburg im Breisgau, Germany; 2 Department for General and Visceral Surgery, Medical Center – University of Freiburg, Faculty of Medicine, University of Freiburg, Freiburg, Germany; 3 Department for General, Visceral and Vascular Surgery, University Hospital Jena, Jena, Germany; 4 Research Program “Else-Kröner-Forschungskolleg AntiAge”, University Hospital Jena, Jena, Germany; 5 Quality Management, Medical Center – University of Freiburg, Faculty of Medicine, University of Freiburg, Freiburg, Germany

**Keywords:** Kidney transplantation, Renal failure, Immunosuppression, Graft-unrelated surgery

## Abstract

**Background:**

Physicians are faced with a growing number of patients after renal transplantation undergoing graft-unrelated surgery. So far, little is known about the postoperative restitution of graft function and the risk factors for a poor outcome.

**Methods:**

One hundred one kidney transplant recipients undergoing graft-unrelated surgery between 2005 and 2015 were reviewed retrospectively. A risk analysis was performed and differences in creatinine, GFR and immunosuppressive treatment were evaluated. Additional, a comparison with a case-matched non-transplanted control group were performed.

**Results:**

Preoperative creatinine averaged 1.88 mg / dl [0.62–5.22 mg / dl] and increased to 2.49 mg / dl [0.69–8.30 mg / dl] postoperatively. Acute kidney failure occurred in 18 patients and 14 patients had a permanent renal failure. Significant risk factors for the development of postoperative renal dysfunction were female gender, a preoperative creatinine above 2.0 mg / dl as well as a GFR below 40 ml / min and emergency surgery. Patients with tacrolimus and mycophenolate mofetil treatment showed a significant lower risk of renal dysfunction than patients with other immunosuppressants postoperatively. Contrary to that, the risk of patients with cyclosporine treatment was significantly increased. Transplanted patients showed a significantly increased rate of postoperative renal dysfunction.

**Conclusions:**

The choice of immunosuppressant might have an impact on graft function and survival of kidney transplant recipients after graft-unrelated surgery. Further investigations are needed.

**Electronic supplementary material:**

The online version of this article (10.1186/s12882-019-1358-2) contains supplementary material, which is available to authorized users.

## Background

During the past decades, kidney transplantation has become a safe therapy for end-stage renal disease [1]. The growing number of patients living with a transplanted kidney brings new challenges like the perioperative management of graft-unrelated surgery to physicians [2]. A typical postoperative complication is acute renal failure, which leads to an increased morbidity and mortality of non-transplanted patients [3] and assumably also of transplanted patients. Besides the risk of acute postoperative renal failure, operating transplanted patients also bears the risk of disturbing the sensitive balance of immunosuppression. Transplanted patients live under immunosuppressive therapy, which has a lot of side effects [4] and interacts with other medical therapies and interventions [5]. The adjustment of immunosuppression walks a fine line between graft rejection and nephrotoxicity [6]. In addition to steroids, the most common immunosuppressants for kidney transplant recipients are tacrolimus and cyclosporine. While tacrolimus and cyclosporine differ in their intracellular binding characteristics, their immunosuppressive properties result from inhibition of calcineurin [6]. Calcineurin dephosphorylates a transcription factor of T-lymphocytes and enhances the immune response [7]. Despite a similar efficacy of tacrolimus and cyclosporine regarding immunosuppression, tacrolimus has been preferred clinically because of a better patient outcome [8]. So far, little is known about the graft function after graft-unrelated surgery and the impact of individual immunosuppression on postoperative graft function. Therefore, we investigated the postoperative graft outcome and mortality of kidney-transplanted patients after graft-unrelated surgery.

## Methods

This was a retrospective monocentric and controlled cohort study of kidney transplant recipients and healthy controls, reported in line with the STROCSS criteria [9]. Data was obtained as previously described [10]. All kidney transplant recipients with a preserved graft function, who were treated at the Department for General and Visceral Surgery of the University Medical Hospital Freiburg between 2005 and 2015 were screened for eligibility and, if eligible, compared to a case-matched control of a non-transplanted patient.

GFR was calculated via CKD-EPI equation. The occurrence of renal dysfunction was noted on the basis of the discharge documents. Acute kidney failure was defined as an increase in creatinine level (reference range 0.51–0.95 mg / dl) by more than 0.3 mg / dl in 48 h or as an oliguria (< 0.5 ml / kg / h) for more than six hours.

IBM SPSS® (version 23.0) was used for exploratory data analysis. Results were checked for normal distribution. Evaluation of group differences was carried out by a T-test as an analysis of two independent groups. A chi-squared test was utilized to test for trends and significance and compare groups of categorical data. Differences with a *p*-value < 0.05 were considered as statistically significant. See Additional file 1 for definition of subgroups.

Study was approved by the ethical committee of the Medical Faculty of the University of Freiburg (EK: 203/17) and was performed according to the principles of the declaration of Helsinki. It was registered in an approved primary register of the WHO (DRKS00015440).

## Results

From January 2005 to December 2015 a total of 1535 kidney transplant recipients were admitted to the Department of General and Visceral Surgery at the Medical Center of the University of Freiburg. Out of these, 101 patients underwent abdominal or abdominal wall surgery and were included in our study (main reasons for exclusion of patients were graft-related surgery and dialysis-depended renal failure).

The meantime since renal transplantation was 15.8 years [± 9.3 years, range 2.8–37.6 years]. The vast majority of patients were transplanted once (mean: 1.29 transplants, maximum of four transplants). The main diagnosis that led to transplantation was chronic glomerulonephritis (*n* = 38; 38%), followed by cystic kidney disease (*n* = 30; 30%). The mean age of included patients was 59 years [range 41–77 years], two thirds of the patients (*n* = 66; 65%) underwent elective surgery.

Overall, 20 patients out of 101 (20%) died postoperatively. The leading causes of death were sepsis (*n* = 14, 13 with abdominal and one with pulmonary sepsis), intractable hemorrhagic shock (n = 3, one due to a pulmonary embolism and two due to gastrointestinal bleedings), cardiovascular complications (*n* = 2) and progress of neoplastic disease (n = 1).

### Perioperative renal dysfunction

Acute kidney failure occurred in 18 of the 101 patients (18%). More than 60% of the patients with acute kidney failure (*n* = 11) had demand on acute dialysis. Overall, 14 patients (14%) developed a permanent renal failure with need of long-time dialysis.

In the subgroup of patients who died during the postoperative hospital stay (*n* = 20), 65 % had acute renal failure, and half of them also required dialysis.

Preoperative creatinine averaged 1.88 mg / dl [0.62–5.22 mg / dl] and increased to 2.49 mg / dl [0.69–8.30 mg / dl] on the first postoperative day. The calculated preoperative GFR averaged 43 ml per minute [8.74–110.30 ml / min] (vs. postoperative GFR 32 ml /min [6.50–101.74 ml / min]). The surviving patients (*n* = 81) were discharged with an averaged creatinine of 1.70 mg / dl [0.24–6.0 mg / dl] and a GFR of 53.85 ml / min [9.14–146.38 ml / min].

### Risk factors for postoperative renal dysfunction (Table 1)

Patients whose preoperative creatinine was higher than 2.0 mg / dl suffered more frequently from postoperative acute renal failure (*p* = 0.026). Similar to that, patients with a GFR lower than 40 ml / min showed significant higher rates of acute renal failure postoperatively (*p* = 0.040). Female patients suffered more frequently from acute and permanent kidney failure (see Table 1). After emergency surgery (compared to elective cases) patients suffered more frequently from acute kidney failure (*p* = 0.009) and had a higher need for dialysis (*p* = 0.005). We found no influence of the extent of surgery (major vs. minor), localization of operation (extra- vs. intra-abdominal), number of transplantations and time since transplantation on the development of renal dysfunction (see Table 1).

**Table 1:** Risk factor analysis of postoperative renal failure in 101 renal-transplant patients undergoing abdominal or abdominal wall surgery

Parameter	n	Dialysis-dependent Renal failure, n (%)	p	Acute renal Failure, n (%)	p	Permanent Renal failure, n (%)	p
Age							
< 60 years	54	4 (7%)	0.228	8 (15%)	0.397	5 (9%)	0.151
> 60 years	47	7 (15%)		10 (21%)		9 (19%)	
Creatinine preoperative							
< 2.0 mg / dl	64	6 (9%)	0.746	7 (11%)	**0.026**	7 (11%)	0.382
> 2.0 mg / dl	35	4 (11%)		10 (29%)		6 (17%)	
Extent							
major	49	7 (14%)	0.288	12 (25%)	0.089	10 (20%)	0.065
minor	52	4 (8%)		6 (12%)		4 (8%)	
Gender							
male	63	5 (8%)	0.220	7 (11%)	**0.023**	5 (8%)	**0.027**
female	38	6 (16%)		11 (29%)		9 (24%)	
GFR preoperative							
> 40 ml / min	45	3 (7%)	0.244	4 (9%)	**0.040**	4 (9%)	0.201
< 40 ml / min	54	7 (13%)		13 (24%)		9 (17%)	
Location							
intraabdominal	78	8 (10%)	0.706	15 (19%)	0.496	11 (14%)	0.897
abdominal wall	23	3 (13%)		3 (13%)		3 (13%)	
Time since transplantation							
< 10 years	34	1 (3%)	0.068	3 (9%)	0.092	2 (6%)	0.098
> 10 years	67	10 (15%)		15 (22%)		12 (18%)	
Timing							
emergency	35	8 (23%)	**0.005**	11 (31%)	**0.009**	8 (23%)	0.057
elective	66	3 (5%)		7 (11%)		6 (9%)	
Transplanted							
Once	78	8 (10%)	0.706	11 (14%)	0.072	9 (12%)	0.213
More than once	23	3 (13%)		7 (30%)		5 (22%)	

Bold entries are significant

### Influence of immunosuppression on mortality and renal dysfunction (Table 2)

Nearly all patients (*n* = 90) received steroids preoperatively. Slightly more than half of the patients (*n* = 55, 55%) were treated with tacrolimus (45 combined with Mycophenolate mofetil (MMF)). 32% (*n* = 32) of the patients were treated with cyclosporine (16 combined with MMF). Further two patients had combination of cyclosporine and azathioprine. Five patients were treated with sirolimus. One patient each had immunosuppression with combination of tacrolimus and cyclosporine, of tacrolimus and azathioprine or a single treatment with everolimus or basiliximab. Due to the low frequency of patients with other immunosuppressants, we focused on the three most common immunosuppressant regimens for further subgroup analysis of the influence of immunosuppressive therapy on postoperative renal function and mortality (see Table 2). The combination of tacrolimus and MMF (*n* = 45) was associated with the lowest rates of renal dysfunction and mortality. The highest rates were found in tacrolimus only treated patients (*n* = 10) and in patients with cyclosporine and MMF (*n* = 16).

**Table 2:** Influence of the three most common preoperative immunosuppressive medications on risk of renal failure and mortality

Immunosuppressant	n	Dialysis-dependent Renal failure, n (%)	p	Acute renal Failure, n (%)	p	Permanent Renal failure, n (%)	p	Mortality	p
Tacrolimus									
no	46	9 (20%)	**0.009**	12 (27%)	**0.041**	10 (22%)	**0.032**	14 (31%)	**0.012**
yes	55	2 (4%)		6 (11%)		4 (7%)		6 (11%)	
only	10	2 (20%)	**0.002**	3 (30%)	**0.034**	3 (30%)	**0.002**	4 (40%)	**0.001**
combined with MMF	45	0 (0%)		3 (7%)		1 (2%)		2 (4%)	
Cyclosporine									
no	69	4 (6%)	**0.06**	8 (12%)	**0.016**	6 (8%)	**0.027**	11 (16%)	0.153
yes	32	7 (22%)		10 (31%)		8 (25%)		9 (28%)	
only	16	3 (18%)	0.780	5 (31%)	1.000	3 (19%)	0.564	4 (25%)	0.780
combined with MMF	16	4 (25%)		5 (31%)		5 (31%)		5 (31%)	
Mycophenolat mofetil									
no	34	6 (18%)	0.121	9 (27%)	0.106	7 (21%)	0.163	10 (29%)	0.084
yes	67	5 (8%)		9 (13%)		7 (10%)		10 (15%)	
…and Tacrolimus	45	0 (0%)	**0.001**	3 (7%)	**0.015**	1 (2%)	**0.001**	2 (4%)	**0.001**
…and Cyclosporin	16	4 (25%)		5 (31%)		5 (31%)		5 (31%)	

Bold entries are significant

The preoperative creatinine of patients with tacrolimus was on average 1.8 mg / dl (±0.7 mg / dl) and the early postoperative creatinine was 2.5 mg / dl (±1.3 mg / dl). Those patients were discharged with a creatinine of 1.67 mg / dl (±0.8 mg / dl). The preoperative creatinine of patients with cyclosporine was on average 1.93 mg / dl (±0.9 mg / dl), and the early postoperative creatinine was 2.6 mg / dl (±1.3 mg / dl). Patients under cyclosporine were discharged with a creatinine of 1.88 mg / dl (±1.0 mg / dl). The differences of creatinine and GFR between the patients with tacrolimus and with cyclosporine were not significant.

### Comparison with control group (Table 3)

We were able to find a case-matched non-transplanted control for 84 of above mentioned 101 transplanted patients. Due to the performed surgical treatment it was not possible to find a suitable case-matched control for 17 patients (see also methods and Additional file 1). They were not considered for further case-matched analysis.

**Table 3:** Case-control-comparison: Description of patients and course of creatinine and GFR (Pre- and postoperative creatinine was not measured in all patients)

	Transplanted (*n* = 84)	Control (*n* = 84)	p
Age [years, mean ± SD]	59.0 ± 9.0	60.5 ± 15.5	0.349
Gender [male/female %]	61.9/38.1	56.0/44.0	0.444
Dialysis-dependent renal failure, n [%]	8 (9.0)	0 (0)	**0.004**
Acute renal failure, n [%]	15 (17.9)	2 (2.4)	**0.001**
Permanent renal failure, n [%]	11 (13.1)	0 (0)	**0.001**
Preoperative	Transplanted (*n* = 82)	Control (*n* = 83)	p
Creatinine [mg / dl, mean ± SD]	1.93 ± 0.88	1.04 ± 1.36	**< 0.001**
GFR^a^ [ml / min / 1.73m^2^ ± SD]	43.29 ± 22.22	84.08 ± 23.63	**< 0.001**
1. Postoperative day	Transplanted (n = 83)	Control (*n* = 74)	p
Creatinine [mg / dl, mean ± SD]	2.57 ± 1.28	1.12 ± 0.73	**< 0.001**
GFR^a^ [ml / min / 1.73m^2^, mean ± SD]	32.05 ± 18.35	75.24 ± 25.64	**< 0.001**
Discharge	Transplanted (n = 82)	Control (*n* = 79)	p
Creatinine [mg /dl, mean ± SD]	1.78 ± 0.94	0.90 ± 0.43	**< 0.001**
GFR^a^ [ml / min/ 1.73m^2^, mean ± SD]	53.85 ± 26.55	90.94 ± 23.13	**< 0.001**
After 6 months	Transplanted (*n* = 58)	Control (*n* = 44)	p
Creatinine [mg / dl, mean ± SD]	2.05 ± 0.90	1.12 ± 0.69	**< 0.001**
GFR^a^ [ml / min / 1.73m^2^, mean ± SD]	41.40 ± 21.84	76.64 ± 26.58	**< 0.001**

*SD* Standard deviation, ^a^GFR was calculated via CKD-EPI equation

Bold entries are significant

**Fig. 1:**
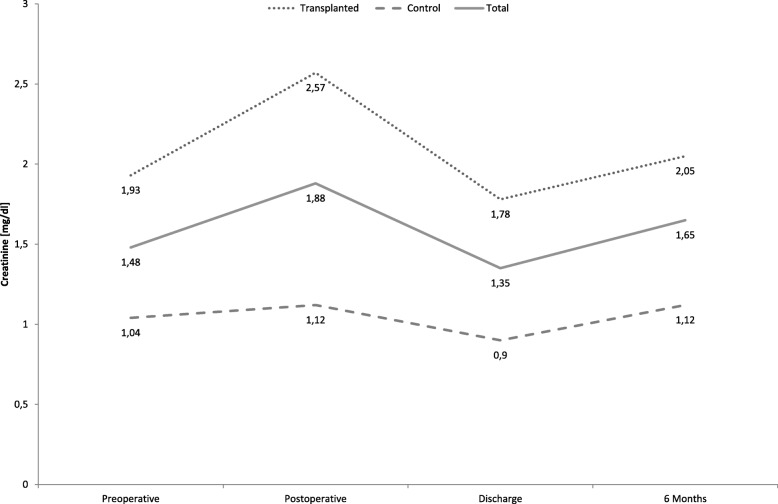
Course of creatinine

**Fig. 2:**
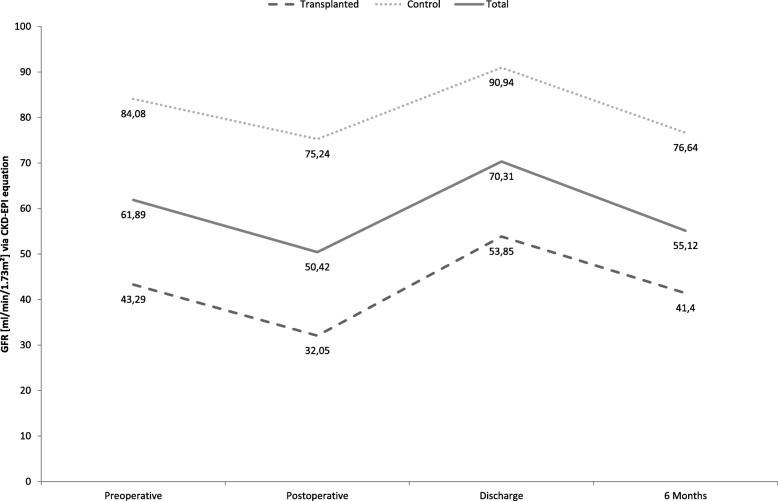
Course of GFR

Descriptive statistics, course of creatinine and GFR as well as the incidence of renal dysfunction of 84 renal transplant recipients and 84 control patients are shown in Table 3. Transplanted patients had a significant higher creatinine (*p* <  0.01, shown in Figs. 1 and 2) and transplanted patients had a significantly higher rate of acute (18% vs. 2%, *p* = 0.001) and permanent (13% vs. 0%, *p* = 0.001) renal failure postoperatively, compared to non-transplanted patients. Additionally, the rate of postoperative dialysis was significantly increased in transplanted patients (9% vs. 0%, *p* = 0.004).

## Discussion

Our study evaluated the graft outcome of 101 renal transplant recipients, providing new insights into the outcome of transplanted patients undergoing graft-unrelated surgery. It is not surprising that kidney-transplanted patients have an increased creatinine in comparison with non-transplanted patients. However, the increased rate of postoperative kidney failure and mortality in transplanted patients is striking. So far, recent literature shows only results of low numbers of transplant recipients or single case studies. Sharma et al. investigated the outcome of 36 renal or liver transplant recipients undergoing cardiac surgery and compared the results with non-transplanted patients. Three patients in the transplant group had demand on dialysis compared to one patient in the control group [11]. Reshef et al. reported the results of 37 solid organ transplant recipients after emergency surgery of the colon and found a renal failure in four patients (vs. one patient in non-transplanted control group) [12]. Contrary to that, Kaluza et al. studied the kidney function of 54 transplanted patients (kidney, kidney-pancreas) after various graft-unrelated surgical procedures and concluded that kidney function remained stable in all patients [13] and Rivas et al. reported no renal complications after laparoscopic colectomy of three transplanted patients [14]. Nyame et al. reported the case of one patient after kidney-pancreas-transplantation, who underwent anterior pelvic exenteration without perioperative renal complications [15]. The results of the other studies are inhomogeneous and due to small sample sizes hard to evaluate.

However, regarding our results we assume that kidney transplant recipients are at a clearly increased risk for postoperative renal dysfunction and death. We could demonstrate that patients with preoperatively worse renal function, conditions requiring emergency surgery and, possibly, female gender were risk factors for impaired renal function postoperatively. A kidney-friendly perioperative treatment, which focuses at the preservation of graft function, is essential for a good graft outcome. Due to the results of our risk analysis special attention should be paid to patients with a limited graft function preoperatively and with a longer time since transplantation. Interestingly, female patients in our study showed a higher risk for postoperative renal dysfunction. Even if gender differences in postoperative complication rates are often discussed, we found no plausible explanation for this observation.

To our knowledge, the study is the first to investigate the postoperative graft function of kidney transplant recipients after graft-unrelated surgery in relation to the chosen immunosuppression. The results suggest that the individual immunosuppressant may influence the postoperative graft outcome. Several publications of the recent years reported advantages of tacrolimus over cyclosporine regarding nephrotoxicity, graft rejection and side effects. Krämer et al. performed a six-month randomized controlled trial and evaluated data of 286 patients with tacrolimus treatment vs. 271 patients with cyclosporine treatment [8]. During 24 month of follow-up the composite endpoint consisting of acute rejection, death or graft loss occurred significantly more frequently in patients with cyclosporine treatment. Other studies showed a negative effect of cyclosporine on renal function and blood pressure compared to tacrolimus [16–18]. Therefore, a negative postoperative renal outcome of patients with cyclosporine seems plausible. Interestingly, in our study tacrolimus only treated patients had the worst outcome, which is contrary to what one might expect. We tried to find a reason for this observation and analyzed the patients in detail. All patients were treated with steroids and one had an additional therapy with azathioprine. 3 patients died due to an abdominal sepsis, one had a fulminant pulmonary embolism with following intractable bleeding. Just one patient had emergency surgery. Deceased patients had also a renal dysfunction. We also analyzed gender, age and comorbidities, but we found no plausible explanation for our observation and assume that it might be a consequence of the small sample size of only 10 patients. Furthermore, it is surprising that just the combination of tacrolimus and MMF showed a supposedly protective effect on renal function and mortality. Contrary to that, the combination of cyclosporine and MMF was worse than tacrolimus and MMF and also worse than cyclosporine only treatment. Comparing patients with MMF and without MMF, we found merely a positive trend for the outcome of MMF treated patients. As above mentioned, just the combination with tacrolimus reached the level of statistical significance. It has to be considered that this might reflect only the harmful effect of other immunosuppressants and is not related to beneficial effects of tacrolimus and MMF. In the end it is just a comparison between nephrotoxic substances. Nevertheless, the results suggest that the chosen immunosuppressant might be important for the postoperative renal outcome. Documentation errors and lack of randomization always limit the results of retrospective evaluation. Due to that and to the exploratory character of our study, the results must be interpreted with care. Future prospective studies have to investigate whether it might be helpful to shift patients with cyclosporine and MMF treatment to tacrolimus and MMF before elective surgery to prevent postoperative renal dysfunction.

## Conclusion

The choice of immunosuppressant might have an impact on graft function and survival of kidney transplant recipients after graft-unrelated surgery, but further investigations are needed.

## Additional file

Additional file 1: Whole strategy of creation of control group, searched encryptions and definitions. (DOCX 42 kb)

## Abbreviations

GFR: Glomerular filtration rate
MMF: Mycophenolate mofetil

## References

[1] Wolfe RA, Ashby VB, Milford EL, Ojo AO, Ettenger RE, Agodoa LY (1999). Comparison of mortality in all patients on dialysis, patients on dialysis awaiting transplantation, and recipients of a first cadaveric transplant. N Engl J Med.

[2] Matas AJ, Gillingham KJ, Humar A, Kandaswamy R, Sutherland DER, Payne WD (2008). 2202 kidney transplant recipients with 10 years of graft function: what happens next?. Am J Transplant.

[3] Jones DR, Thomas Lee H (2008). Perioperative renal protection. Best Pract Res Clin Anaesthesiol.

[4] Bamoulid J, Staeck O, Halleck F, Khadzhynov D, Paliege A, Brakemeier S (2016). Immunosuppression and results in renal transplantation. Eur Urol Suppl.

[5] Naesens M, Kuypers DRJ, Sarwal M (2009). Calcineurin inhibitor nephrotoxicity. Clin J Am Soc Nephrol.

[6] Issa N, Kukla A, Ibrahim HN (2013). Calcineurin inhibitor nephrotoxicity: a review and perspective of the evidence. Am J Nephrol.

[7] Rusnak F, Mertz P (2000). Calcineurin: form and function. Physiol Rev.

[8] Krämer BK, Montagnino G, del Castillo D, Margreiter R, Sperschneider H, Olbricht CJ (2005). Efficacy and safety of tacrolimus compared with cyclosporin a microemulsion in renal transplantation: 2 year follow-up results. Nephrol Dial Transplant.

[9] Agha RA, Borrelli MR, Vella-Baldacchino M, Thavayogan R, Orgill DP, Pagano D (2017). The STROCSS statement: strengthening the reporting of cohort studies in surgery. Int J Surg.

[10] Lederer A-K, Haffa D, Martini V, Huber R, Makowiec F, Fichtner-Feigl S (2019). Surgical outcomes of renal transplant recipients after abdominal surgery not connected with transplantation. A retrospective case-control study. Int J Surg.

[11] Sharma R, Hawley C, Griffin R, Mundy J, Peters P, Shah P (2013). Cardiac surgical outcomes in abdominal solid organ (renal and hepatic) transplant recipients: a case-matched study. Interact Cardiovasc Thorac Surg.

[12] Reshef A, Stocchi L, Kiran RP, Flechner S, Budev M, Quintini C (2012). Case-matched comparison of perioperative outcomes after surgical treatment of sigmoid diverticulitis in solid organ transplant recipients versus immunocompetent patients. Color Dis.

[13] Kałuża B, Ziobrowski I, Durlik M (2012). Surgical procedures not connected with transplantation in patients after kidney or kidney and pancreas transplant with stable function of graft. Pol Przegl Chir.

[14] Rivas H, Martínez J-L, Delgado S, Lacy AM (2004). Laparoscopic assisted colectomies in kidney transplant recipients with colon cancer. J Laparoendosc Adv Surg Tech A.

[15] Nyame YA, Nandanan N, Greene DJ, Krishnamurthi V, Haber G-P (2016). Robotic anterior pelvic Exenteration for bladder Cancer in patient with previous kidney-pancreas transplantation. Urology..

[16] Klein IHHT, Abrahams A, van Ede T, Hené RJ, Koomans HA, Ligtenberg G (2002). Different effects of tacrolimus and cyclosporine on renal hemodynamics and blood pressure in healthy subjects. Transplantation..

[17] Radermacher J, Meiners M, Bramlage C, Kliem V, Behrend M, Schlitt HJ (1998). Pronounced renal vasoconstriction and systemic hypertension in renal transplant patients treated with cyclosporin a versus FK 506. Transpl Int.

[18] Taylor DO, Barr ML, Radovancevic B, Renlund DG, Mentzer RM, Smart FW (1999). A randomized, multicenter comparison of tacrolimus and cyclosporine immunosuppressive regimens in cardiac transplantation: decreased hyperlipidemia and hypertension with tacrolimus. J Heart Lung Transplant.

