# Supplementary material for: The impact of immunosuppression on postoperative graft function after graft-unrelated surgery: a retrospective controlled cohort study
Source: BMC Nephrol. 2019 May 16;20:170. doi: 10.1186/s12882-019-1358-2 (PMC6521488; doi:10.1186/s12882-019-1358-2)
Supplement: Supplementary file 1 — Whole strategy of creation of control group, searched encryptions and definitions. (DOCX 42 kb) [file 12882_2019_1358_MOESM1_ESM.docx]

**Appendix 1: Whole strategy of creation of control group**

Protocol for creating the control group, created by DH and AKL after consultation of LK, 08-23-2017:

Due to the fact that OPS encryption of surgical procedures leads to an amount of potential control patients, we decided to choose randomly an operation date between 01-01-2005 and 12-31-2015 to limit the search results.

**First step: Creation of random list for operation dates**

- Creation of a random generator with Microsoft Excel®
- Formula: DATE (2005; 1; 1) + Random Area (0; 4016)
  (4016 is the number of days between 01-01-2005 and 12-31-2015)
- List was created by AKL under control of DH on 08-23-2017

**Second step: Searching the control patient**

Search was performed by DH under control of AKL and LK

1. Opening of electronic patient management system “Prometheus”
2. Starting search function, limit for general surgery as well as for transplanted patients and children
3. Entering the desired OPS encryption
4. Entering a randomly created date as start time for search, end time was 1 year after the randomly created date
5. Activating the “Diagnosis / Therapy” view
6. Start searching
7. Checking the first result for eligibility
- If the first patient was not eligible for inclusion, documentation of reasons and checking of the second one, if the patient was also not eligible for inclusion, documentation of reasons and checking of the third one (and so on)
- If search was unsuccessful, new search with another randomly created date

Inclusion criteria:
- type of operation and as well the urgency of operation (emergency / elective) have to correspond with operation in the transplanted group
- not transplanted
- preserved kidney function

Exclusion criteria:
- patient had an organ transplantation
- insufficient renal function with requirement of dialysis
- organ removal of brain dead patients
- re-operation of a complication
- patient already in control group

**Appendix 2: Searched encryptions**“OPS”: The Operations and Procedure Key (OPS) is the official classification used to encrypt operations, procedures and general medical procedures in Germany. The OPS is an adaptation of the International Classification of Procedures in Medicine (ICPM) of the World Health Organization (WHO).
(see http://www.dimdi.de/static/en/klassi/ops/index.htm for more information)

| 5-536.4  5-465.1  5-437.23  5-511.0  5-511.1  5-455.72  5-455.62  5-455.42  5-455.41  5-454.20  5-454.1  5-456.00  5-455.75  5-455.77  5-530.3  5-534.3  5-413.10  5-470.0  5-466  5-536.0  5-462  5-524.2  5-455.71  5-464.23  1-694 | 5-460.20  5-455.71  5-484.51  5-467.00  5-455.21  5-455.71  5.501.00 + 5-511  5.501.00 + 5-511  5-502 + 5-501.10  5-455.61 + 5-511  5-466 + 5-511  5-502.0 + 5-511  5-536.4 + 5-536.1  5-455.71 + 5-462.1 + 5-471.0  5-466 + 5-455  5-541 + 5-47  5-455.42  4-455.01  5-523.2  5-502 - 5-511 |
| --- | --- |

**Appendix 3: Definitions**

**Minor surgery:**

Adhesiolysis, creation of ascending stoma, appendectomy, cholecystectomy, diagnostic laparoscopy, exploratory laparotomy (without further resection), suture of duodenum, restoration of ileostomy, hernia (inguinal, navel, scar) repair with or without mesh, revision of stoma and removal of retroperitoneal hematoma. Stoma revisions and hernia repairs were also defined as an extra-abdominal operation

**Major surgery:**

Wedge resections and non-anatomic resections of the liver, anatomic resections of the liver, right or left hemihepatectomy, segmental resections of small bowel, all types of colectomy and rectal resection, gastrointestinal continuity restoration surgery, esophagectomy, gastrectomy, pseudocystojejunostomy, pancreatic necrosectomy, cystic kidney nephrectomy, splenectomy, pancreaticoduodenectomy

**Extra-abdominal:**

All hernia repairs as well as stoma revisions
